# Adipocytes fuel gastric cancer omental metastasis *via* PITPNC1-mediated fatty acid metabolic reprogramming: Erratum

**DOI:** 10.7150/thno.101689

**Published:** 2024-09-05

**Authors:** Yujing Tan, Kelin Lin, Yang Zhao, Qijing Wu, Dongping Chen, Jin Wang, Yanxiao Liang, Jingyu Li, Jiazhu Hu, Hao Wang, Yajing Liu, Shuyi Zhang, Wanming He, Qiong Huang, Xingbin Hu, Zhiqi Yao, Bishan Liang, Wangjun Liao, Min Shi

**Affiliations:** 1 Department of Oncology, Nanfang Hospital, Southern Medical University, Guangzhou, China; 2 Department of Radiation Oncology, Affiliated Cancer Hospital & institute of Guangzhou Medical University, Guangzhou, China; 3 Department of Abdominal Surgery, Affiliated Cancer Hospital & institute of Guangzhou Medical University, Guangzhou, China; 4 Department of Pathology, Guangzhou First People's Hospital, Guangzhou, China; 5 Department of Pathology, Zhujiang Hospital, Southern Medical University, Guangzhou, China; 6 Department of Oncology, Guangzhou Panyu Central Hospital, Guangzhou, China; 7 Department of Pathology, Guangzhou Panyu Central Hospital, Guangzhou, China

The authors apologize that the original version of the above article contains errors that need to be corrected. Incorrect images in Figure 3F and Figure 4C and Figure 5E were inserted in figure assembly. The corrected versions of the figures appear below. The authors declare that these amendments do not change the results or conclusions of the paper. The authors sincerely apologize to the Journal and its readers for the confusion this may have caused.

## Figures and Tables

**Figure 3:**
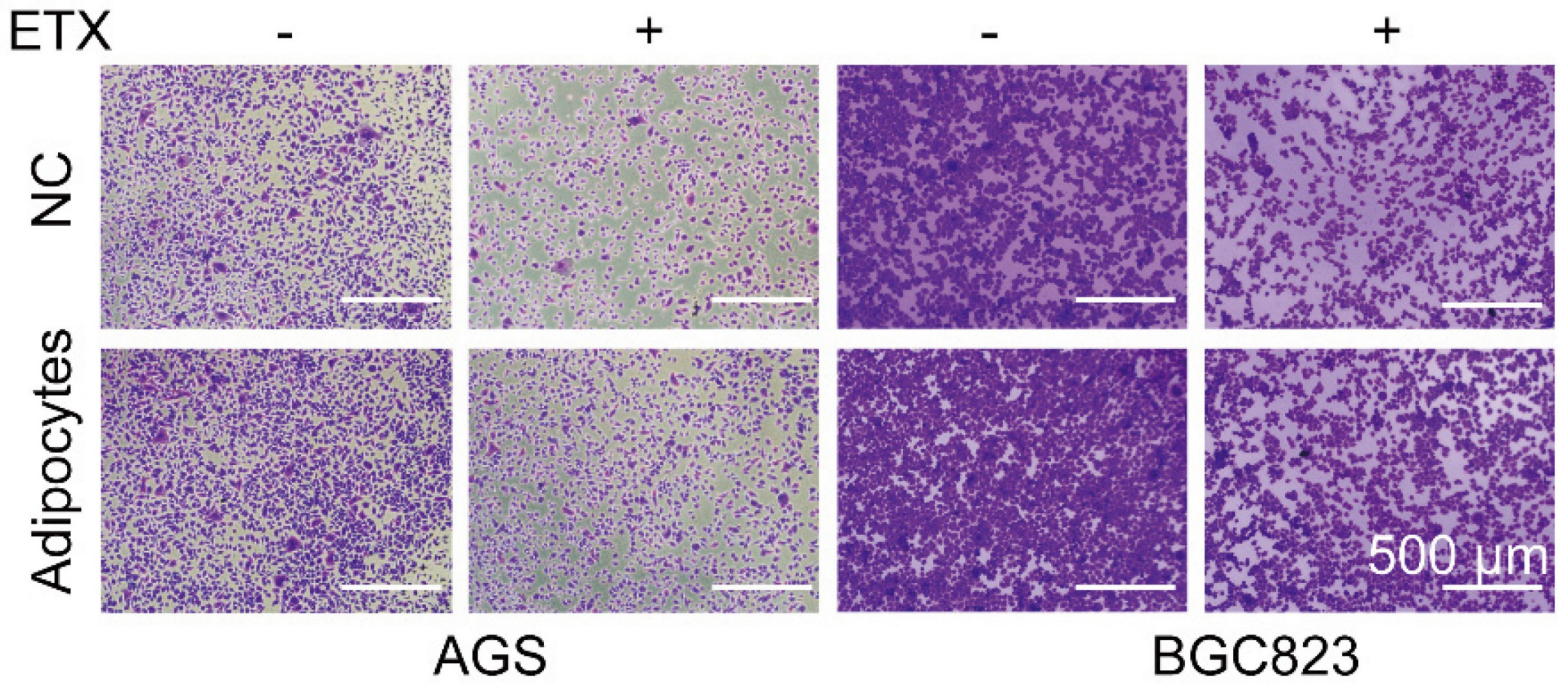
Correct image of Figure 3F.

**Figure 4:**
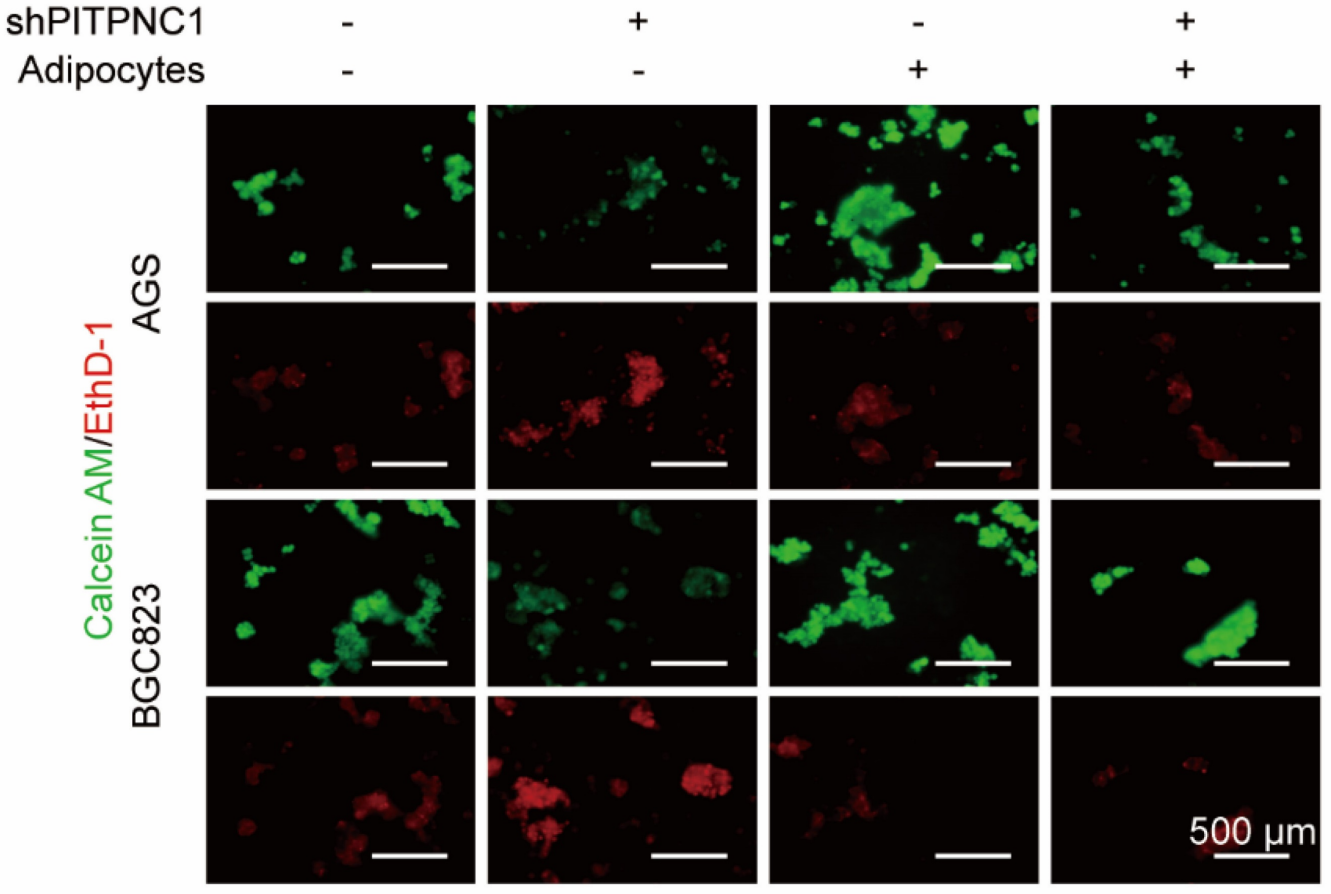
Correct image of Figure 4C.

**Figure 5:**
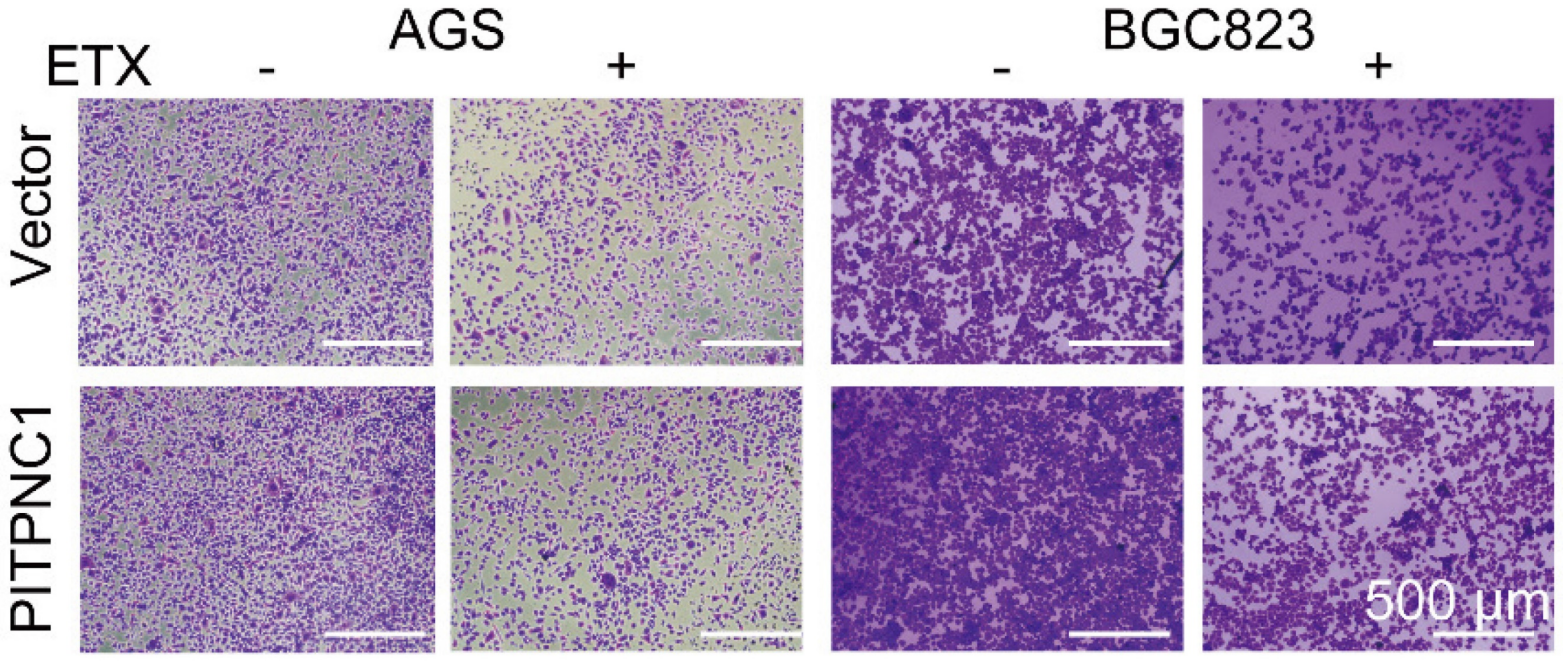
Correct image of Figure 5E.

